# Mechanism of methyltransferase like 3 in epithelial-mesenchymal transition process, invasion, and metastasis in esophageal cancer

**DOI:** 10.1080/21655979.2021.1994721

**Published:** 2021-11-27

**Authors:** Xuyang Liang, Zhimei Zhang, Lu Wang, Shuxian Zhang, Ling Ren, Shouying Li, Jing Xu, Shengxiang Lv

**Affiliations:** Department of Gastroenterology, The Affiliated Lianyungang Hospital of Xuzhou Medical University/The First People’s Hospital of Lianyungang, Lianyungang, China; Department of Gastroenterology, The First Affiliated Hospital of Kangda College, Nanjing Medical University/The First People’s Hospital of Lianyungang, Lianyungang, China; Department of Gastroenterology, Lianyungang Clinical College of Nanjing Medical University/The First People’s Hospital of Lianyungang, Lianyungang, China

**Keywords:** Esophageal cancer, METTL3, m6A modification, DGCR8, pri-miR-20a-5p, miR-20a-5p, NFIC, epithelial-mesenchymal transition

## Abstract

Methyltransferase like 3 (METTL3) has been identified to serve as a definitive inducer in cancer progression. This study sought to analyze the regulatory mechanism of METTL3 in epithelial-mesenchymal transition (EMT), invasion, and metastasis in esophageal cancer (ESCA). The METTL3 expressions in cancer tissues and cells were detected with extensive analysis of its correlation with clinical baseline data. The cells were transfected with sh-RNA-METTL3 and microRNA (miR)-20a-5p mimic, followed by evaluation of invasion, migration, and EMT. The N6-methyladenosine (m6A) level and enrichment of DiGeorge Critical Region 8 (DGCR8) and m6A were observed. The binding relationship between miR-20a-5p and Nuclear Factor I-C (NFIC) was verified, followed by Pearson correlation analysis. A subcutaneous tumor formation assay was conducted prior to observation of lung metastases. Our results revealed that METTL3 was highly expressed in ESCA patients and associated with severe lymph node involvement and distant metastasis. METTL3 downregulation radically inhibited the invasiveness, migration, and EMT. METTL3 elevated the miR-20a-5p expression via improving m6A modification. METTL3 inhibition downregulated the miR-20a-5p expression. Moreover, miR-20a-5p upregulation facilitated ESCA cell invasiveness and migration by targeting NFIC transcription. METTL3 inhibition suppressed tumor growth and lung metastasis *in vivo*. Overall, METTL3 promoted m6A modification and the binding of DGCR8 to miR-20a-5p to further elevate the miR-20a-5p expression and inhibit NFIC transcription, thus promoting EMT, invasion and migration.

## 1. Introduction

Esophageal cancer (ESCA) is classified as the aberrant proliferation and metastasis of squamous cell carcinoma and adenocarcinoma [1]. Statistically, ESCA is regarded as the sixth leading cause of cancer associated mortality worldwide with a 5-year survival rate of less than 18% [2]. Physicological factors such as the degree of invasiveness and metastasis are vital causes of the high mortality among ESCA patients [3]. Currently, surgical resection has been the definitive cornerstone in ESCA treatment [1]. Unfortunately, the associated vigorous metastases among half of ESCA patients make surgical intervention ineffective [4]. Epithelial-mesenchymal transition (EMT) is a carcinogenic process that can rapidly facilitate the transformation of primary tumors into invasive malignancies [5]. Accumulating evidence has identified the therapeutic effects of targeting EMT in ESCA treatment [6]. Therefore, an investigated is necessitated for the identification of novel targets to prevent EMT, invasion, and metastasis so as to improve the therapeutic outcomes of ESCA treatment.

N6-methyladenosine (m6A), has been the chief component in regulating the internal modification in eukaryotic messenger RNA to influence the gene expression [7]. Methyltransferase like 3 (METTL3) is an RNA methyltransferase complex that can radically catalyze m6A modification with vital functionality in various biological processes of malignancies, such as proliferation, angiogenesis, invasion, and metastasis [8]. Notably, existing evidence identified the ability of METTL3 to promote proliferation, invasion, and metastasis in ESCA [9,10]. Additionally, METTL3 could promote tumor growth by regulating m6A modification to facilitate EMT progression in various types of cancers [11,12]. However, the molecular mechanism of METTL3-mediated m6A modification in ESCA warrants extensive investigation.

MicroRNAs (miRNAs) are small noncoding RNAs ranging over 21–25 nucleotides in length with clinical application as potential therapeutic targets of ESCA [13]. An existing study has demonstrated that METTL3 could improve m6A modification to elevate the expression of miRNA [14]. Fundamentally, miR-20a is an oncogene with a prominent overexpression in ESCA patients [15]. Moreover, miR-20a-5p could evidently to promote invasion, EMT, metastasis, and tumor growth in various cancers, such as triple-negative breast cancer and colorectal cancer [16,17]. However, whether METTL3 could significantly modulate the miR-20a-5p expression via m6A modification in the development of ESCA remain elusive.

Moreover, a targeting relationship has been identified between miR-20a-5p and the nuclear factor I-C (NFIC) protein via database prediction and dual-luciferase reporter assay in the present study. NFIC protein is a part of the NFI family, which is primarily downregulated in most types of cancers and associated with tumor suppressors [18]. In ESCA, NFIC elicits an inhibitory property in EMT to suppress proliferation and metastasis [19]. Yet, the regulatory relationship between miR-20a-5p and NFIC in ESCA remains unidentified and warrants thorough investigation.

In light of the aforementioned findings, we speculated that METTL3 could regulate miR-20a-5p/NFIC signaling to manipulate EMT, invasion, and metastasis in ESCA. Hence, the present study sought to evaluate the functional mechanism of METTL3 in ESCA *in vitro* and *in vivo*, hoping to provide a novel strategy for advances in ESCA treatment.

## 2. Methods and materials

### 2.1 Ethics statement

The animal experiment complied with the Guidelines for the Use and Management of Laboratory Animals [20] and was conducted with approval of the Laboratory Animal Ethics Committee of The Affiliated Lianyungang Hospital of Xuzhou Medical University. All participants provided informed written consent prior to participation.

### 2.2 Acquisition of ESCA tissues *[21]*

The cancer tissue and paracancerous normal tissue specimens were harvested from a total of 60 ESCA patients who underwent pathological resection in the oncology department of The Affiliated Lianyungang Hospital of Xuzhou Medical University from a period between March 2016 and March 2020. The tissue specimens were preserved using liquid nitrogen and stored in a refrigerator at −80°C. All patients were validated with ESCA after histopathological examination, with no family history of malignancy or associated medical history, and received no treatment prior to this study.

### 2.3 Cell culture and treatment *[22]*

Human ESCA cell lines ECA109, EC9706, KYSE150, TE-1, and TE-10 and normal esophageal epithelial cell line HEEC (Shanghai Huiying Biological Technology Co., Ltd, Shanghai, China) were cultured using Dulbecco’s modified Eagle medium (DMEM) supplemented with a combination of 10% fetal bovine serum, 100 U/mL penicillin and 100 μg/m streptomycin (Gibco, Carlsbad, CA, USA) in a thermostatic incubator at 37°C with 5% CO_2_. The METTL3 expression was knocked down in cells using the sh-METTL3 recombinant lentivirus. Plasmid construction and virus packaging were conducted by GenePharma Co., Ltd (Shanghai, China) with empty plasmid serving as control. The cells in the logarithmic growth phase were seeded into 6-well plates (2 × 10^5^ cells/well) for subsequent infection with sh-METTL3 lentivirus for 48 h strictly according to the total amount of virus at 40 multiplicity of infection/cell. The cells stably transfected with sh-METTL3 were screened for subsequent experiments. Next, the well-grown cells were seeded into 6-well plates (2 × 10^5^ cells/well) for 24 h-incubation, followed by transfection with miR-20a-5p mimic using Lipofectamine 2000 (11,668–019, Invitrogen, Carlsbad, CA, USA) for an overexpression of miR-20a-5p with mimic NC as control. After 48 h, the transfected cells were isolated for subsequent experimentation.

### 2.4 Reverse transcription quantitative polymerase chain reaction (qRT-PCR) *[15]*

PCR primers were screened using the Bio-Rad software (Bio-Rad, Hercules, CA, USA) and synthesized by Shanghai Sangon Biotech Co., Ltd (Shanghai, China). The total RNA content was extracted using the RNA Extraction kits (Promega, Madison, WI, USA). The 1 μg RNA was reverse transcribed into complementary DNA using the PrimerScript RT Master Mix (Takara, Dalian, China). Next, qRT-PCR was performed using SYBR Premix Ex Taq (Takara) in ABI 7500 (ABI, Foster City, CA, USA). The relative gene expression was qualified based on the 2^−ΔΔCT^ method with GAPDH serving as control of METTL3 and NFIC and U6 serving as control of miR-20a-5p. PCR primers are shown in Table 1.

**Table 1.** Primer sequence

Name of primer	Sequences
miR-20a-5p-F	TAAAGTGCTTATAGTGCAGGTAG
miR-20a-5p-R	TGGTGTCGTGGAGTCG
U6-F	CTCTTCGGGCAGCATATACT
U6-R	ACGCTTCACACATATACT
METTL3-F	ATGAGTCTTTAGGTGACTGCT
METTL3-R	TCCATGCAAGCATCAATTTCA
NFIC-F	ATGAGTTCCACCCGTTCATCG
NFIC-R	GAGACCGAAGCAGGTGGATCA
GAPDH-F	GGAGCGAGATCCCTCCAAAAT
GAPDH-R	GGCTGTTGTCATACTTCTCATGG

### 2.5 Immunofluorescent staining *[23]*

The cells were seeded into 96-well plates (5 × 10^3^ cells/well). Upon attaining 80–90% confluence, the cells were fixed using 4% paraformaldehyde buffer and permeabilized using 1%Triton X-100, followed by membrane blockade with 5% skim milk for 30 min. Next, the cells were cultured with the corresponding primary antibodies N-cadherin (at a dilution ratio of 1:200, ab18203, Abcam) and E-cadherin (at a dilution ratio of 1:200, ab40772, Abcam) at 37°C for 2 h and then with the secondary goat anti-rabbit Ig (immunoglobulin) G (at a dilution ratio of 1:5000, ab6721, Abcam) at 37°C for 1 h. Next, the cells were stained with dihydrochloride at 37°C for 5 min prior to observation and documentation under a fluorescence microscope (Olympus, Tokyo, Japan).

### 2.6 Transwell assays *[19]*

In strict accordance with the provided instructions of Transwell assay, cell invasion, and migration potentials were evaluated using 24 well-Transwell chambers (BD Biosciences, San Jose, CA, USA). For invasion assay, 50 mg/L Matrigel was diluted at the proportion of 1/8 and coated at the bottom of the chamber. In the migration assay, the chamber was not coated with Matrigel. The basolateral chamber was supplemented with 600 μL of complete medium and the apical chamber was supplemented with 200 μL cell suspension, followed by a regimen of 24 h-incubation at 37°C. Next, the cells in the apical chamber were removed using cotton swabs. The invasive or migratory cells were fixed with methanol at 4°C for 30 min and stained with 0.1% crystal violet solution at 37°C for 20 min, followed by observation under an inverted microscope (Nikon Corporation, Tokyo, Japan)

### 2.7 m6A quantification *[24]*

The total RNA content was separated from 1 × 10^6^ cells using the TRIzol reagent (Invitrogen). The RNA m6A level was determined using the m6A RNA Methylation Quantification kit, and the absorbance value was analyzed using the SpectraMax Plus384 microplate Reader (Molecular Device, Sunnyvale CA, USA). Briefly, 150 µg of the total RNA content was dissolved in 500 mL of Rnase-free water and mixed with Biotinylated-Oligo (Promega) at room temperature for 10 min, followed by the addition of Streptavidin-Paramagnetic Particles (Promega). RNA containing polyA^+^ was separated from the solution using magnetic beads with biotin-streptavidin conjugate. The polyA^+^ enriched RNA was fragmented using the RNA Fragmentation Buffer (Millipore, Bedford, MA, USA). Dynabeads containing 5 µg of anti-m6A (at a dilution ratio of 1:1000; ab230356; Abcam) were used to bind to RNA fragments containing m6A methylation prior to the elution of RNA fragments from beads and overnight precipitation at 4°C. The enrichment of m6A in miR-20a-5p or pri-miR-20a-5p was detected using the provided primer-probe sets (Bogu Co., Ltd, Shanghai, China).

### 2.8 RNA co-immunoprecipitation *[24]*

RNA co-immunoprecipitation was conducted using the Magna RIP RNA Binding Protein Immunoprecipitation kit (Millipore). Briefly, the cells were lysed, and mixed with anti-m6A (at a dilution ratio of 1:1000, ab230356, Abcam), anti-DiGeorge Critical Region 8 (DGCR8) (at a dilution ratio of 1:1000, ab191875, Abcam) or IgG (at a dilution ratio of 1:2500, ab150077, Abcam). RNA bound to the antibody was pulled down using protein A/G magnetic beads, followed by quantification using real-time qPCR.

### 2.9 Dual-luciferase reporter assay *[19]*

The binding sites between miR-20a-5p and NFIC were analyzed through the Starbase website (http://starbase.sysu.edu.cn/). Next, the NFIC 3ʹUTR sequence containing the binding sites and mutation sites was cloned into the luciferase vector pGL3 (Promega) to construct the NFIC-wild type and NFIC- mutant type plasmids. The 293 T cells (ATCC, Manassas, VA, USA) were seeded into a 6-well plate for a regimen of 24 h-incubation. Subsequently, the constructed plasmids with mimic NC or miR-20a-5p mimic (Shanghai Genechem Co., Ltd., Shanghai, China) (miRNA-mimic 30 nM) were co-transfected into the 293 T cells using Lipofectamine 2000 (11,668–019, Invitrogen). After 24 h, the luciferase activity was assessed using the Dual-Lucy Assay Kit (Solarbio, Beijing, China). Each cell experiment was conducted 3 times independently.

### 2.10 Subcutaneous tumor formation assay *[25,26]*

Male nude mice (age: 4–6 weeks, weight: 22–22 g) provided by the Southern Medical University, (Guangzhou, China, Approval No: SCXK(Guangdong) 2016–0041) were housed in specific pathogen environment at 22–24°C and 50–60% humility under a 12-h light/dark cycle, with ad libitum access to food and water. The nude mice were classified into 3 groups with 18 mice per group: the control group, the sh-NC group, and the sh-METTL3 group. Well-grown ECA109 cells, sh-METTL3-transfected ECA109 cells, sh-NC-transfected ECA109 cells were re-suspended in phosphate buffer saline (PBS) solution and then subcutaneously injected (1 × 10^7^cells/mouse) into the right abdomen (N = 6) or injected into mice via the caudal veins (N = 6). For the subcutaneously injected mice, the tumor volume was evaluated every 7 days according to the following formula: V = *ab*^2^/2 (a: the longest diameter of the tumor; b: the shortest diameter of the tumor). After 30 days, the subcutaneously injected mice were euthanatized using an intraperitoneal injection of 1% pentobarbital (200 mg/kg). Next, the tumors were isolated and weighted. Subsequently, the tumor tissues were used to extract the protein and RNA content. Caudal vein-injected mice were euthanatized after 50 days. The lung tissues were harvested from the chest followed by formaldehyde fixing and paraffin embedding. Next, the specimens were divided into 5 µm sections, followed by hemotoxylin and eosin (HE) staining to precisely observe the size and number of lung metastatic nodules.

### 2.11 Western blotting *[26]*

The cells were lysed using the Radioimmunoprecipitation Assay lysis buffer containing 1% phenylmethanesulfonyl fluoride prior to centrifugation at 14,000 g and 4°C for 30 min to extract the total protein content, followed by quantification using the Bradford Method Protein Assay Kit (Beyotime, Shanghai, China). Next, the protein content was boiled for 5 min, cooled on ice, and centrifuged for 30 s. The supernatant liquid was isolated using sodium dodecyl sulfate-polyacrylamide gel and transferred onto polyvinylidene fluoride membranes at 100 V. After a membrane blockade with 5% skim milk at room temperature, the membranes were cultured with the corresponding primary antibodies at 4°C for 12 h. Subsequently, the membranes were rinsed with tris buffered saline tween twice for incubation with luciferase-labeled goat anti-rabbit IgG (at a dilution ratio of 1:2500, ab6721, Abcam) at room temperature for 1 h. After 3 rinses, the membranes were visualized using the enhanced chemiluminescence and photographed with a membrane scanner. The included primary antibodies were as follows: N-cadherin (at a dilution ratio of 1:1000, ab76011, Abcam), E-cadherin (at a dilution ratio of 1:1000, ab40772, Abcam), and GAPDH (at a dilution ratio of 1:1000, ab9485, Abcam).

### 2.12 Statistical analysis

A combination of the SPSS21.0 software (IBM Corp, Armonk, NY, USA) and GraphPad Prism 8.0 software (GraphPad Software Inc., San Diego, CA, USA) were used for data analysis and graphing. The experimental data comprised of enumeration and measurement data. The pairwise comparisons of enumeration data were analyzed using the chi-square test. Measurement data were presented as mean ± standard deviation (SD). Comparisons of measurement data among multiple groups were analyzed using one-way or two-way analysis of variance (ANOVA), followed by Tukey’s multiple comparison test. The pairwise comparisons of measurement data were analyzed using the *t* test. Pearson correlation analysis was used for analysis of correlations between two continuous variables. The *P* value was obtained from two-tailed tests. In all statistical references, a value of *P* < 0.05 was considered to be statistically significant.

## 3. Results

This study sought to determine the role of m6A modification of METTL3 in EMT, invasion, and metastasis of ESCA. Our results elucidated that m6A modification of METTL3 could facilitate ESCA progression via regulation of the miR-20a-5p/NIFC axis. In this study, we chose ESCA cell lines and patient samples to validate our hypothesis. Initially, two types of ESCA cells were transfected with sh-METTL3 to determine the functional role of METTL3 in EMT, invasion, and migration of ESCA, m6A, and METTL3 expression patterns were determined, with analysis of the interaction between METTL3 and miR-20a-5p. In our study, the miR-20a-5p expression pattern was increased in the ESCA cells to evaluate the role of miR-20a-5p in ESCA and the binding relationship of ESCA and NIFC was verified via dual-luciferase reporter assay. Moreover, we validated the role of METTL3 in ESCA *in vivo*.

### 3.1 METTL3 was increased in ESCA

**Figure 1.**
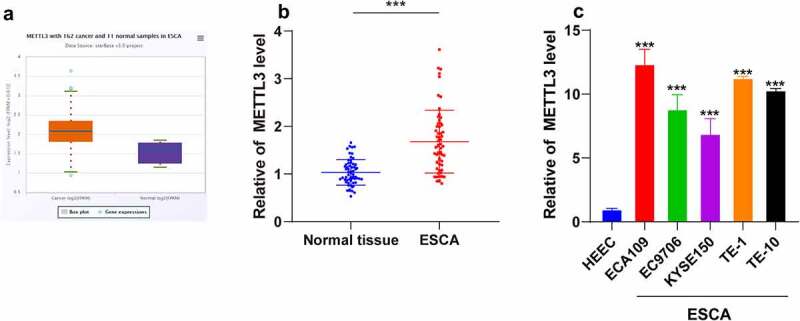
METTL3 was upregulated in ESCA. ESCA tissues and normal paracancerous tissues were collected. ESCA cell lines (ECA109, EC9706, KYSE150, TE-1, and TE-10) were selected with normal HEECs as the control. A: METTL3 expression pattern in ESCA patients was analyzed through the Starbase website (http://starbase.sysu.edu.cn/); B: METTL3 expression pattern in ESCA tissues and normal paracancerous tissues were detected via qRT-PCR; C: METTL3 expression pattern in different ESCA cell lines and HEECs were detected via qRT-PCR. Cell experiment was conducted 3 times independently. Enumeration data in figure B were analyzed using the *t* test. Measurement data in figure C were represented as mean ± SD and analyzed using one-way ANOVA, followed by Tukey’s multiple comparison test. *** *P* < 0.001

METTL3 is a methyltransferase that can modulate the initiation and progression of various types of cancers [27]. To determine the function of METTL3 in ESCA, the METTL3 expression pattern in cancer samples and normal samples was analyzed by the Starbase website (http://starbase.sysu.edu.cn/). Our results demonstrated that METTL3 was upregulated in the 162 ESCA samples (Figure 1a). Next, we detected the METTL3 expression pattern in cancer tissues and normal paracancerous tissues from 60 ESCA patients and the results of qRT-PCR revealed an markedly elevated METTL3 expression pattern in ESCA tissues compared to the normal paracancerous tissues (*P* < 0.001; Figure 1b). Based on the median value of METTL3 expression pattern (2.1, 1.49 ~ 2.79), the patients were divided into a high group (H-METTL3) and a low group (L-METTL3). The clinical baseline data (Table 2) revealed that the H-METTL3 group had more serious lymph node involvement (P = 0.0202) with more distant metastases (P = 0.004) compared with the L-METTL3 group, which shared no correlation with the age and gender of patients (*P* > 0.05). Next, the differential expression pattern of METTL3 in human ESCA cell lines ECA109, EC9706, KYSE150, TE-1, and TE-10 and human normal esophageal epithelial cells (HEECs) was detected via qRT-PCR and the results showed an elevated METTL3 expression pattern in different human ESCA cell lines relative to HEECs (*P* < 0.001; Figure 1c). Altogether, a high expression pattern of METTL3 was identified in ESCA and associated with cancer metastasis.

**Table 2.** Clinical baseline data

**Characteristics**		**H-METTL3** **N = (30)**	**L-METTL3** **N = (30)**	***X*^2^/t**	***P* value**
Age (years)		52.39 ± 3.81	50.42 ± 4.34	1.868	0.0668
Gender	Male	19	14	1.684	0.1945
	Female	11	16		
T stage	T_1_~ T_2_	17	24	3.774	0.0521
	T_3_~ T_4_	13	6		
N stage	N_1_	8	11	7.807	**0.0202**
	N_2_	7	14		
	N_3_	15	5		
M stage	M_0_	7	18	8.297	**0.004**
	M_1_	23	12		

Tumor-Node-Metastasis Staging : T: Tumor size and/or primary location; N: Lymph node involvement; M: Distant metastasis.

### 3.2 METTL3 promoted EMT, invasion, and migration of ESCA cells

**Figure 2.**
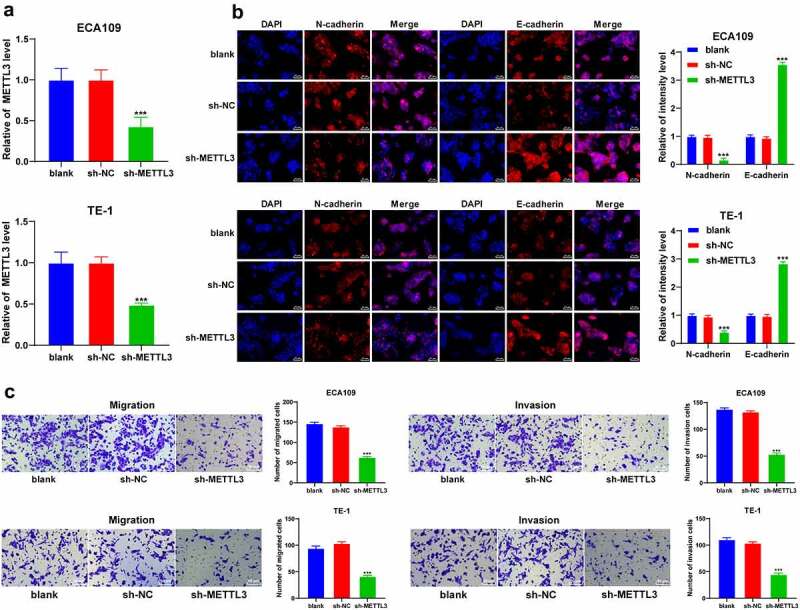
METTL3 promoted EMT, cell invasion, and migration in ESCA cells. ECA109 and TE-1 cells were transfected with sh-METTL3 with sh-NC as the control. EMT, cell migration, and invasion were observed. A: METTL3 expression pattern in ECA109 and TE-1 cells was detected via qRT-PCR; B: Positive expression patterns of N-cadherin and E-cadherin were detected via immunofluorescence; C: Cell invasion and migration potentials were detected via Transwell assays; Cell experiment was conducted 3 times independently. All data were measurement data and represented as mean ± SD. Data in figures A and C were analyzed using one-way ANOVA and data in figure B were analyzed using two-way ANOVA. After analysis, data were verified by Tukey’s multiple comparison test. *** *P* < 0.001

To explore the role of METTL3 in EMT, invasion, and migration in ESCA cells, the ECA109 and TE-1 cells with a relatively high expression pattern of METTL3 were selected in strict accordance with the results in Figure 1c, followed by transfection of sh-METTL3 to downregulate the METTL3 expression pattern (*P* < 0.001; Figure 2a). After METTL3 downregulation, the positive expression pattern of N-cadherin was reduced, while the positive expression pattern of E-cadherin was increased (*P* < 0.001; Figure 2b). Moreover, the results of Transwell assays showed inhibited cell invasion and migration potentials after METTL3 downregulation (*P* < 0.001; Figure 2c). Briefly, METTL3 could promote EMT, invasion and migration of ESCA cells.

### 3.3 METTL3 facilitated m6A modification in pri-miR-20a-5p to upregulate miR-20a-5p expression

**Figure 3.**
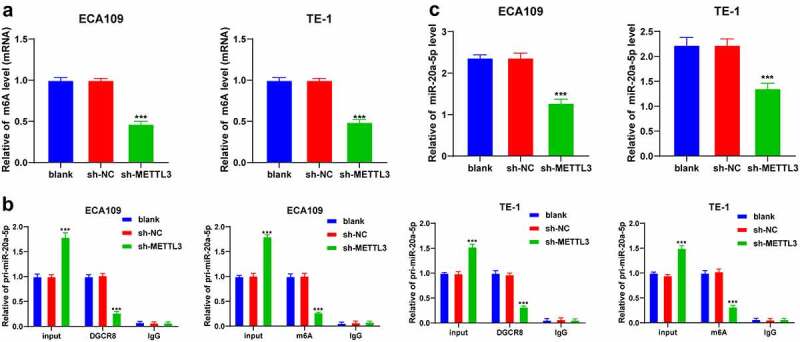
METTL3 promoted m6A methylation in pri-miR-20a-5p to upregulate miR-20a-5p expression pattern. ECA109 and TE-1 cells were transfected with sh-METTL3 with sh-NC as the control. The role of METTL3 in the epigenetic regulation of miR-20a-5p was observed. A: m6A modification level in total RNA of ECA109 and TE-1 cells was detected via m6A assay kits; B: Enrichment of DGCR8 and m6A in pri-miR-20a-5p in ECA109 and TE-1 cells was detected via RNA co-immunoprecipitation assay; C: the miR-20a-5p expression pattern in ECA109 and TE-1 cells was detected via qRT-PCR. Cell experiment was conducted 3 times independently. All data were measurement data and represented as mean ± SD. Data in figures A and C were analyzed using one-way ANOVA and data in figure B were analyzed using two-way ANOVA. After analysis, data were verified by Tukey’s multiple comparison test. *** *P* < 0.001

Existing research has demonstrated the functionality of METTL3 as a methyltransferase to induce modification of m6A in RNA [28]. We detected the level of m6A from the total RNA content of ESCA cells and identified a considerably reduced m6A modification level in ESCA cells after METTL3 downregulation (*P* < 0.001; Figure 3a). DGCR8 specifically recognizes and incorporates pri-miRNAs to promote splicing into the precursor of microRNA, and m6A methylation facilitates the recognition and binding to pri-microRNA by DGCR8, thereby elevating the expression patterns of mature miRNAs [14]. Moreover, an elevated miR-20a expression pattern has been previously identified in ESCA [15]. Hence, we speculated an explicit role of miR-20a-5p in ESCA via m6A modification by METTL3. To validate the postulation, RNA co-immunoprecipitation assay was performed in ESCA cells using the DGCR8 and m6A antibodies. Our results showed that the loss of METTL3 reduced the enrichment of DGCR8 and m6A in pri-miR-20a-5p (*P* < 0.001; Figure 3b). Additionally, METTL3 downregulation reduced the miR-20a-5p expression pattern in ESCA cells (*P* < 0.001; Figure 3c). The preceding results elucidated that METTL3 promoted m6A modification level in pri-miR-20a-5p to improve the binding of DGCR8 to pri-miR-20a-5p, thus upregulating the miR-20a-5p expression.

### 3.4 miR-20a-5p upregulation reversed the effects of METTL3 downregulation on inhibiting EMT, invasion, and migration of ESCA cells

**Figure 4.**
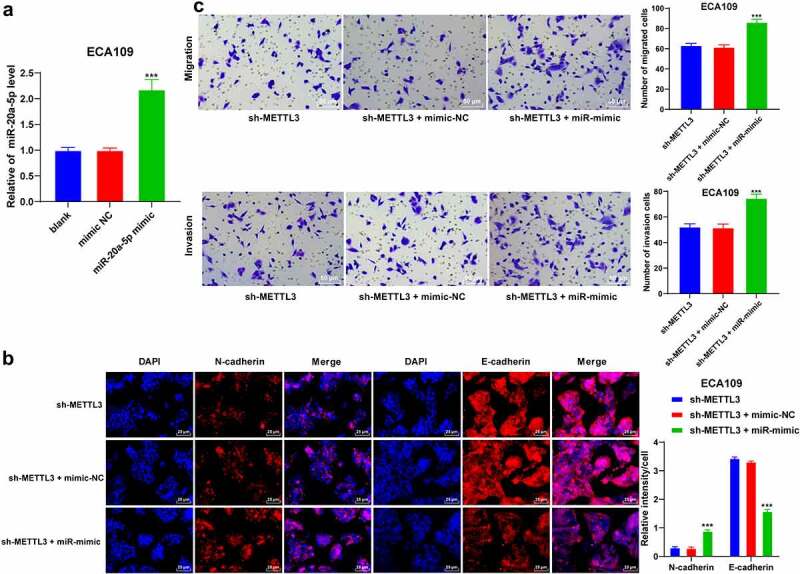
miR-20a-5p promoted EMT, invasion, and migration of ESCA cells. ESCA cells with a poor expression pattern of METTL3 were transfected with miR-20a-5p mimic, with mimic NC as the control. EMT process, cell proliferation, migration, and invasion were observed. A: miR-20a-5p expression pattern was detected via qRT-PCR; B: Positive expression patterns of N-cadherin and E-cadherin were detected via immunofluorescence; D: Cell invasion and migration potentials were detected via Transwell assays; Cell experiment was conducted 3 times independently. All data were measurement data and represented as mean ± SD. Data in figures A and C were analyzed using one-way ANOVA and data in figure B were analyzed using two-way ANOVA. After analysis, data were verified by Tukey’s multiple comparison test. *** *P* < 0.001

To validate whether METTL3 regulates miR-20a-5p in the ESCA cells, sh-METTL3-treated ESCA cells were transfected with the miR-20a-5p mimic to upregulate the miR-20a-5p expression pattern (*P* < 0.001; Figure 4a). After miR-20a-5p upregulation, the positive expression pattern of N-cadherin was elevated while the positive expression pattern of E-cadherin was reduced (*P* < 0.001; Figure 4b). The results of Transwell assays showed that miR-20a-5p upregulation improved the migration and invasion potentials of ESCA cells (*P* < 0.001; Figure 4c). Collectively, the preceding results demonstrated that miR-20a-5p upregulation annulled the inhibitory role of METTL3 downregulation in EMT, cell invasion, and migration of ESCA cells.

### 3.5 miR-20a-5p inhibited NFIC transcription

**Figure 5.**
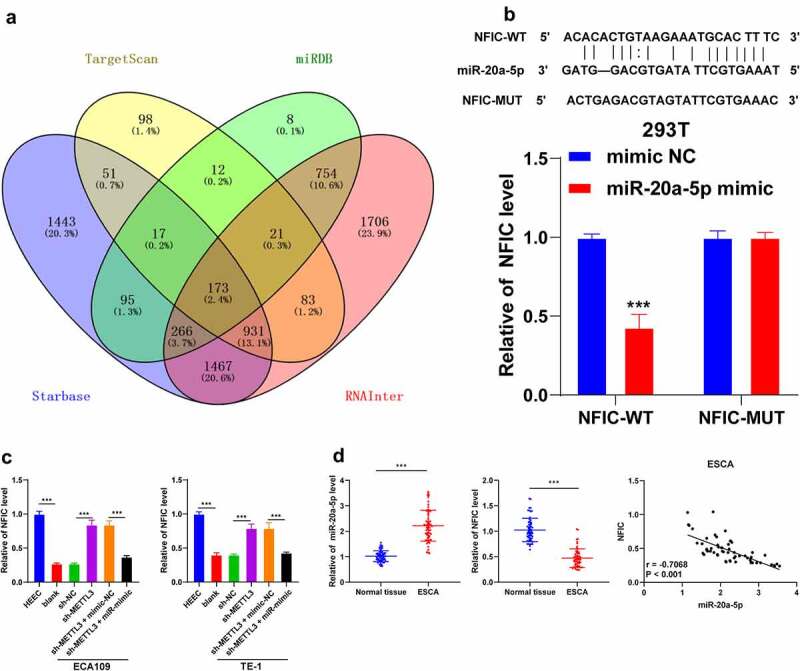
NFIC was a target gene of miR-20a-5p. A: Downstream target genes of miR-20a-5p were predicted through the Starbase (http://starbase.sysu.edu.cn/), Targetscan (http://www.targetscan.org/vert_72/), miRDB (http://mirdb.org/) and RNAInter (http://www.rna-society.org/raid/search.html) and intersections were obtained; B: Binding relationship between miR-20a-5p and NFIC was verified by dual-luciferase reporter assay; C: NFIC expression pattern in different cell groups were detected via qRT-PCR; D: Expression patterns of miR-20a-5p and NFIC in ESCA tissues were detected via qRT-PCR, followed by Pearson correlation analysis. Cell experiment was conducted 3 times independently. Data in figures B and C were measurement data and represented as mean ± SD. Data in figure B were analyzed using two-way ANOVA and data in figure C were analyzed using one-way ANOVA. After analysis, data were checked by Tukey’s multiple comparison test. Data in figure D were enumeration data analyzed using the *t* test, followed by Pearson correlation analysis. *** *P* < 0.001

To further analyze the molecular mechanism of miR-20a-5 in ESCA, the downstream target genes of miR-20a-5p were predicted through a combination of Starbase (http://starbase.sysu.edu.cn/), Targetscan (http://www.targetscan.org/vert_72/), miRDB (http://mirdb.org/) and RNAInter (http://www.rna-society.org/raid/search.html) websites with identification of intersections(Figure 5a). Our researchers focused on the function of NFIC. Previously, NFIC has been demonstrated to play a regulatory role in esophageal squamous cell cancer [19]. The binding relationship between miR-20a-5p and NFIC was verified by dual-luciferase reporter assay in the 293 T cells (*P* < 0.001; Figure 5b). The results of qRT-PCR showed a decreased NFIC transcriptional level in ESCA cells, while it was increased after sh-METTL3 transfection (*P* < 0.001; Figure 5c). Next, we detected the expression patterns of miR-20a-5p and NFIC in 60 pairs of cancer tissues and normal paracancerous tissues, followed by Pearson correlation analysis of miR-20a-5p and NFIC. Our results indicated a highly elevated expression pattern of miR-20a-5p was highly expressed with a weakened expression pattern of NFIC in ESCA tissues (*P* < 0.001; Figure 5d) and they were negatively correlated in the negative ESCA tissues (*r = −0*.4447, *P* = 0.0004, Figure 5d). In conclusion, our results elicited that miR-20a-5p inhibited NFIC transcription in ESCA.

### 3.6 METTL3 downregulation inhibited tumor growth and metastasis in vivo

**Figure 6.**
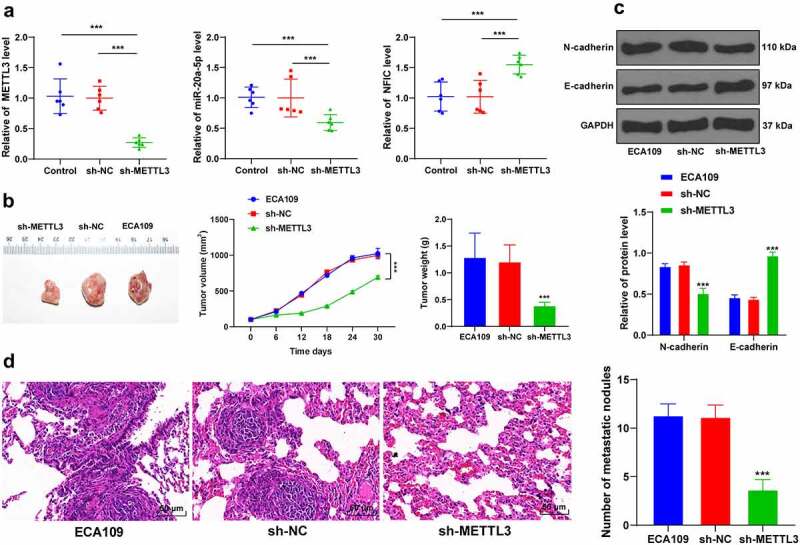
METTL3 downregulation inhibited tumor growth and metastasis *in vivo*. Nude mice were subcutaneously or caudal-vein injected with sh-METTL3-transfected ECA109 cells with sh-NC as the control. A: Expression patterns of METTL3, miR-20a-5p, and NFIC were detected via qRT-PCR; B: Volumes and weights of representative tumors were measured; C: Expression patterns of N-cadherin and E-cadherin were detected via western blot; D: Lung metastases were observed via HE staining. N = 6, all data were measurement data and represented as mean ± SD. Data in figures A, B (tumor weight), D were analyzed using one-way ANOVA, and data in figures B (tumor volume) and C were analyzed using two-way ANOVA. After analysis, data were checked by Tukey’s multiple comparison test. *** *P* < 0.001

To validate the preceding results *in vivo*, the nude mice were subcutaneously injected with the sh-METTL3-transfected ECA109 cells to induce subcutaneous tumor formation. Our results demonstrated that METTL3 and miR-20a-5p were downregulated while the NFIC transcriptional level was upregulated in the tumors (*P* < 0.001; Figure 6a), and METTL3 inhibition had suppressed tumor growth (*P* < 0.001; Figure 6b). Moreover, METTL3 inhibition also reduced the protein level of N-cadherin while it increased the protein level of E-cadherin (*P* < 0.001; Figure 6c). The results of HE staining were illustrative of metastatic nodules in lung tissues, whereas METTL3 inhibition reduced the number of metastatic nodules (Figure 6d). Overall, METTL3 downregulation could inhibit the miR-20a-5p expression pattern and promote NFIC transcription, thereby suppressing the growth and metastasis of ESCA.

## 4. Discussion

ESCA is a highly invasive and metastatic malignancy [3]. The associated developments with the progression of ESCA are EMT, invasion and metastasis [29]. METTL3 is a methyltransferase to primarily enhance m6A modification to facilitate the development of EMT, invasion, and metastasis in various types of cancers [30]. Specifically, a prior study identified that METTL3 overexpression could facilitate the development of ESCA via regulation of oncogenes [31]. In this study, we elucidated that METTL3 could mediate EMT, invasion, and metastasis in ESCA by targeting the miR-20a-5p/NFIC axis.

METTL3 can function as a regulator for chief oncogenes to facilitate tumorigenesis and tumor progression [27]. Initially, our findings identified an increased concentration of METTL3 in 162 ESCA samples, ESCA tissues, and cells. A comprehensive analysis of the clinical baseline data of ESCA patients revealed that the patients with a high expression of METTL3 presented with considerably severe lymph node involvement and distant metastases. The preceding results are in consistency with a previous study highlighting an elevated METTL3 expression in ESCA with increased metastasis [9]. Next, the ECA109 and TE-1 cells with relatively high expression of METTL3 for through analysis were selected for subsequent experimentation. Previously, EMT manifestation was identified with notable molecular alternations, such as elevated N-cadherin and decreased E-cadherin expressions [5]. To determine the role of METTL3 in EMT, invasion, and migration in ESCA cells, we initially silenced the METTL3 expression in ECA109 and TE-1 cells. After silencing METTL3, an elevated E-cadherin positive expression was identified with suppressed N-cadherin positive expression, migration, and invasion potentials. Consistently, an METTL3 overexpression could evidently induce the proliferation, invasion, and migration of ESCA cells via targeting AKT signaling [10]. Moreover, accumulating evidence has elicited the ability of METTL3 to facilitate the EMT process in various types of cancers. For instance, METTL3 can exacerbate EMT in gastric cancer by activation of the zinc finger MYM-type containing 1 signaling [12], while it can also upregulate the JunB proto-oncogene to mediate the progression of EMT in lung cancer [32]. For the currently available literature, our study pioneered the investigation to demonstrate the driving role of METTL3 in EMT in ESCA. Moreover, experimental mice were injected with sh-METTL3-transfected ECA109 cells. Our results showed that METTL3 downregulation had radically suppressed tumor growth and the positive expression of Ki67 with decreased N-cadherin and number of metastatic nodules, and an increased E-cadherin expression. Similarly, an existing study determined the ability of sh-METTL3 to impede the formation of metastatic nodules in colorectal cancer [33]. The *in vivo* results further validated that METTL3 facilitated EMT, invasion, and metastasis in ESCA.

METTL3 is an m6A methyltransferase that can enhance the recognition and binding of DGCR8 to pri-microRNA, thereby increasing the functional expression of mature miRNA [14]. Moreover, existing research identified an elevated miR-20a expression in ESCC patients [15]. In the miR-20a family, miR-20a-5p can serve as a definitive inducer in cancer progression [34]. Therefore, we speculated that METTL3 could increase the miR-20a-5p expression via m6A modification. Our findings implicated that METTL3 silencing reduced the m6A modification level in the total RNA content of ESCA cells along with the enrichment of m6A and DGCR8 in pri-miR-20a-5p. Moreover, METTL3 silencing reduced the miR-20a-5p expression in ESCA cells, indicating that METTL3 increased the m6A modification level in order to facilitate the binding of DGCR8 to pri-miR-20a-5p, thus ultimately elevating the miR-20a-5p expression. We initially investigated the effect of METTL3 regulation on miR-20a-5p in ESCA.

For a comprehensive analysis of the role of miR-20a-5p in ESCA, the ECA109 cells with sh-METTL3 were transfected with miR-20a-5p mimic. Our findings revealed that after miR-20a-5p upregulation, EMT, migration, and invasion potentials were all enhanced. The functional role of miR-20a-5p in the invasion, migration, and EMT in ESCA was consistent with its role in colorectal cancer and triple-negative breast cancer as indicated by previous literature [16,17]. Briefly, the current study initially demonstrated that miR-20a-5p can function as a mediator in EMT and invasion in ESCA.

Subsequently, we investigated the downstream mechanism of miR-20a-5p. Through extensive database prediction and intersections, we focused on NFIC. An existing study identified the ability of NFIC to serve as a crucial transcription factor in esophageal squamous cell cancer [35]. Our results presented with a reduced NFIC transcriptional level in ESCA cells and up-regulation after METTL3 inhibition. Next, the binding relationship between NFIC and miR-20a-5p was verified via dual-luciferase reporter assay, while the Pearson correlation analysis validated the negative correlation between NFIC and miR-20a-5p. Moreover, METTL3 inhibition could increase the NFIC transcriptional level to repress tumor growth and lung metastasis *in vivo*. An existing research determined the inhibitory role of NFIC in the invasion, migration, or metastasis via suppression of EMT in esophageal squamous cell cancer [19]. Similarly, NFIC has been implicated in the treatment of bladder cancer and glioblastomas [36,37]. Altogether, METTL3 elevated the miR-20a-5p expression to inhibit NFIC transcription, thereby facilitating EMT, metastasis, and invasion in ESCA.

## 5. Conclusions

**Figure 7.**
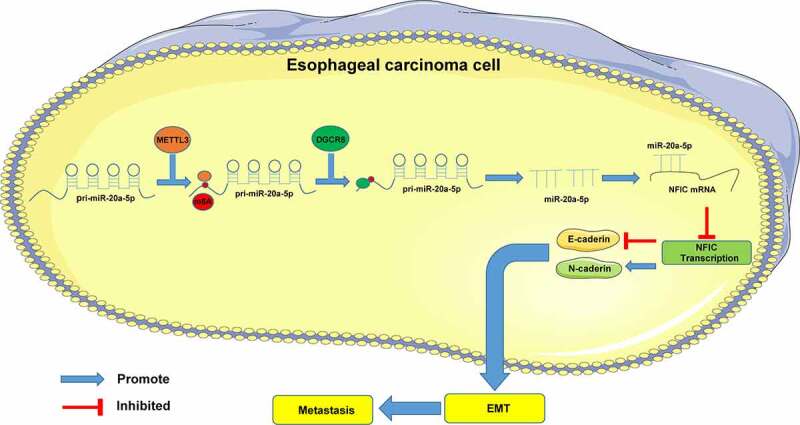
Mechanism of METTL3 in ESCA. METTL3 improved m6A modification level in miR-20a-5p to facilitate the binding of DGCR8 to pri-miR-20a-5p and further to promote the expression pattern of mature miR-20a-5p and inhibit NFIC transcription, thereby promoting EMT, invasion, and metastasis in ESCA

To conclude, our results initially identified that METTL3 by functioning as a methyltransferase can improve the overall m6A level in ESCA cells and the m6A level in miR-20a-5p to facilitate the recognition and binding of DGCR8 to pri-miR-20a-5p and to subsequently promote the expression of mature miR-20a-5p and inhibit NFIC transcription, thereby extensively promoting EMT, invasion, and metastasis in ESCA (Figure 7). However, this study was unable to comprehensively investigate the regulatory role of NFIC in ESCA cells. Moreover, only ECA109 cells were used to establish the transplanted tumor model and lung metastasis model *in vivo*, although it is sufficient to confirm the role of METTL3 on other types of ESCA cell lines. Our future studies will investigate the mechanism of NFIC in ESCA cells with *in vivo* TE-1 tumor model establishment to validate our findings.

## Data Availability

The data that support this study are available from the corresponding author upon reasonable request.

## Research highlights

1. METTL3 is highly expressed in ESCA.
2. METTL3 inhibition suppresses EMT, invasion, and metastasis of ESCA.
3. METTL3 promotes m6A modification in pri-miR-20a-5p to improve miR-20a-5p level.
4. miR-20a-5p inhibits NFIC transcription.
5. METTL3 promotes ESCA progression via targeting the miR-20a-5p/NFIC axis.
